# Extracting Plant Phenology Metrics in a Great Basin Watershed: Methods and Considerations for Quantifying Phenophases in a Cold Desert

**DOI:** 10.3390/s16111948

**Published:** 2016-11-18

**Authors:** Keirith A. Snyder, Bryce L. Wehan, Gianluca Filippa, Justin L. Huntington, Tamzen K. Stringham, Devon K. Snyder

**Affiliations:** 1 USDA-ARS, Great Basin Rangelands Research Unit, Reno, NV 89512, USA; b.wehan@gmail.com; 2 Environmental Protection Agency of Aosta Valley, ARPA Valle d’Aosta, Climate Change Unit, Saint-christophe 11020, Italy; g.filippa@arpa.vda.it; 3 Western Regional Climate Center, Desert Research Institute, Reno, NV 89512, USA; justin.huntington@dri.edu; 4 Department of Agriculture, Nutrition and Veterinary Science, University of Nevada, Reno, NV 89557, USA; tstringham@cabnr.unr.edu (T.K.S.); devonsnyder@cabnr.unr.edu (D.K.S.)

**Keywords:** StarDot cameras, PhenoCam network, pinyon and juniper, sagebrush steppe, semi-arid meadows, camera-based repeat digital photography

## Abstract

Plant phenology is recognized as important for ecological dynamics. There has been a recent advent of phenology and camera networks worldwide. The established PhenoCam Network has sites in the United States, including the western states. However, there is a paucity of published research from semi-arid regions. In this study, we demonstrate the utility of camera-based repeat digital imagery and use of R statistical *phenopix* package to quantify plant phenology and phenophases in four plant communities in the semi-arid cold desert region of the Great Basin. We developed an automated variable snow/night filter for removing ephemeral snow events, which allowed fitting of phenophases with a double logistic algorithm. We were able to detect low amplitude seasonal variation in pinyon and juniper canopies and sagebrush steppe, and characterize wet and mesic meadows in area-averaged analyses. We used individual pixel-based spatial analyses to separate sagebrush shrub canopy pixels from interspace by determining differences in phenophases of sagebrush relative to interspace. The ability to monitor plant phenology with camera-based images fills spatial and temporal gaps in remotely sensed data and field based surveys, allowing species level relationships between environmental variables and phenology to be developed on a fine time scale thus providing powerful new tools for land management.

## 1. Introduction

Studies and observations of plant phenology have been critical to ecology, agricultural production, and understanding multi-trophic interactions. Land managers and producers have been assessing phenophases through field-based surveys for centuries to determine relationships with weather, climate, and proper management strategies. In the last several decades, remotely sensed data has provided various indices of plant vigor at large spatial scales that have provided invaluable information on vegetation cover [1,2,3]. Recent advances in image processing software for land-based repeat digital photography have facilitated user ability to process large amounts of images and the creation of phenology networks to quantify vegetation temporal dynamics in a variety of plant communities around the globe [4,5,6,7,8,9]. Land-based camera vegetation indices have high temporal and spatial resolution (i.e., sub-daily between 1 cm^2^ and 100 cm^2^) and provide unique opportunities to integrate spatial and temporal phenological information for detailed process understanding. For example, land-based sensors can help link point in time field-based measurements with larger scale satellite derived remotely sensed indices, such as from Landsat, to improve monitoring and land management at multiple scales [10]. Long-term monitoring of vegetation phenology may reveal important trends in shifts in the start and end of the growing season, capture patterns of expansion or contraction in groundwater dependent ecosystems, aid in the monitoring of invasive annual grasses with distinct early season phenology, and provide strategies for adaptively managing these ecosystems [11,12].

Multiple land-based camera phenology networks have been detailed [9,13,14], where they analyze time series images acquired from fixed cameras by extracting raw digital numbers (DN) along a relative scale (0–255) of color channels red, green, blue, and/or near infrared (NIR). These per channel DN values are commonly used to calculate various vegetation indices that represent vegetative response and vigor [4,6,15]. These methods have been shown to be quite robust for phenology and gross primary productivity of deciduous forests [16,17,18], and determining fractional snow cover [19]. However, Brown et al. [9] proposed that far less is known about their robustness in temperate and tropical evergreen forests [20], and warm and cold desert environments [21]. Deciduous forests and grasslands are characterized by large seasonal changes in photosynthetic tissue and pronounced changes in color that can be easily detected. Cameras and associated regions of interest (ROIs) are generally pointed at continuous forest canopies, or in the case of the short grassland, at an area with little bare ground [22]. In water limited warm and cold desert environments, seasonal shifts in canopy cover and spectral reflectance can be much more subtle with a substantial fraction of reflectance coming from soil. Increased soil influence on vegetation reflectance is a common issue in environments with sparse vegetation. Therefore, the utility of land-based phenology cameras in arid environments with relatively low vegetation cover remains to be determined.

A large portion of the Great Basin is characterized by: pinyon (*Pinus* spp.) and juniper (*Juniperus* spp.) woodlands, Sagebrush steppe communities, and high elevation meadows [23]. Cheatgrass (*Bromus tectorum*), a prolific invasive annual grass characterized by an early season growing phenology [24] is invading many communities in the Great Basin. Pinyon and juniper (PJ) has expanded into sagebrush steppe systems causing negative ecosystem consequences [25,26,27]. Western juniper (*Juniperus occidentalis*) catchments have been found to have greater snow water equivalent, but on average a nine day earlier melt out, than areas where juniper was removed and sagebrush was the dominant species [28]. Changes in watershed hydrology have the potential to alter groundwater or instream water delivery to riparian meadows thereby impacting the phenophases of meadow communities and the spatial extent of groundwater dependent ecosystems [11,29]. Therefore, quantification of the relationship between plant phenophases and integrative environmental variables such as soil water content and groundwater need to be developed. Active management actions to reduce PJ density and/or decrease the presence of cheatgrass may have impacts on the seasonality of plant available water and plant phenology. Additionally, climate change predictions also suggest that much of the snow dominated areas in the Great Basin may become rain dominated by the mid-21st century [30]. Thus, there is a need to quantify how plant phenology may vary which could have cascading trophic level effects [31,32]. The ability to relate in situ community phenology to field based measurements of environmental variables will ultimately lead to filling critical data needs that cannot be adequately addressed in greenhouse experiments and may provide a monitoring method for rapidly determining ecosystem change. Quantitative estimates of vegetation phenology and vigor should also prove useful for developing adaptive management plans for rangeland vegetation.

The purpose of this study was to determine if these cameras, standardized installation and the *phenopix* package were useful in determining plant phenology of a semi-arid region in the dominant community types of the Central Great Basin. We measured air temperature, soil temperature, soil water content at 20 cm, and groundwater depth in a meadow, to determine if plant phenology was related to water availability and temperature. Specifically, we aimed to determine if there were detectable phenological differences between: (1) wet and mesic meadows; (2) pinyon canopies and juniper canopies and (3) sagebrush underlain by groundwater versus upland sagebrush. Furthermore, we perform pixel by pixel analyses in the sagebrush community type to determine if these analyses might be useful in assessing spatially variable patterns through time.

## 2. Materials and Methods

### 2.1. Study Area

Land-based camera images, plant cover, weather data, soil and groundwater measurements were conducted in Porter Canyon Experimental Watershed (PCEW), which is located on the eastern side of the Desatoya Mountain Range near Austin, NV (39°28′ N; 117°37′ W, 2195 m elevation) and within the Central Great Basin . Mountain block geology of the PCEW is predominantly comprised of rhyolitic welded ash-flow tuffs, lava flows and intrusive rocks, while lowlands are comprised of shallow alluvium and stream deposits. Soil types within the upland sagebrush area are classified as loamy-skeletal, mixed, mesic typic argixerolls, whereas the soils within the mesic meadow area are frigid cumulic haploxerolls, and the wet meadow area are frigid cumulic endoaquolls [33,34]. The PCEW is typical of the Central Great Basin ecoregion, a dry and cold system with the majority of available soil water generally occurring in spring after winter precipitation and snowmelt [35]. Vegetation in the PCEW is characterized by singleleaf pinyon (*Pinus monophylla*) and Utah juniper (*Juniperus osteosperma*) woodlands, sagebrush (*Artemisia* spp.) steppe communities, and valley bottom meadows. Pinyon and juniper (PJ) are native coniferous evergreen trees. Sagebrush is considered a semi-deciduous evergreen shrub as it retains leaves throughout the year. Sagebrush has two set of leaves: a persistent set that grow in spring and summer and are retained through winter; and an ephemeral set of larger leaves that emerge in spring, but are senesced at the height of the summer dry period with last year’s persistent leaves. Meadows are characterized by perennial sedges (*Carex* spp.) and grasses and show seasonal green up and senescence. The long-term average precipitation for the site is 335 mm with approximately 70% of the precipitation in winter months. The monthly average temperatures range from 20.7 °C in July to −0.8 °C in January [36]. Data presented in the current study are from calendar year 2015, with the exception of vegetation surveys which were done in July of 2014 to characterize plant communities.

### 2.2. Camera Sites and Overview

**Figure 1:**
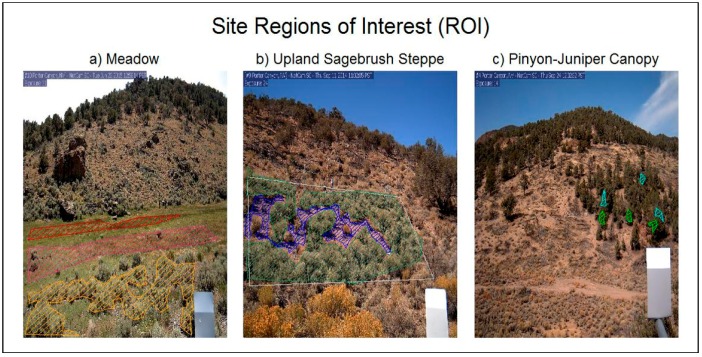
Regions of Interest (ROI) at three site locations. (**a**) Meadow site of wet, mesic and dry meadow sagebrush canopy in red, pink and orange respectively; (**b**) ROI for upland sagebrush steppe in white, canopy in green and interspace in blue; (**c**) Pinyon and juniper canopy site with 3 pinyon ROIs in cyan and 3 juniper ROIs in green.

We used methods and land-based cameras standardized by the PhenoCam Network [4,9] along with the *phenopix* package [37] to evaluate vegetation phenology. The PhenoCam Network has developed a standard protocol when implementing phenology cameras, thus forming a foundation for comparing annual phenology across differing ecoregions. An R statistical program package, *phenopix* developed by Filippa et al. (2016) [38], has greatly improved the processing of images by offering algorithm choices for image filtering, curve fitting, and phenological phase (phenophase) date determination. Land-based cameras were installed in the PCEW at adjacent PJ woodland, sagebrush steppe and meadow sites in October of 2014 at elevations ranging from 2225 m to 2100 m (Figure 1). Methods used for camera data processing and complementary environmental data collection, including detailed vegetation metrics, soil water content, and meteorology measurements, are described below.

### 2.3. Vegetation

Canopy cover and plant density transects were completed for all plant communities in July 2014. These data were collected to identify the primary vegetation in each phenocam site in order to understand which individual species may be driving phenological changes in the imagery. Canopy cover of upland plant communities was measured with a continuous line intercept method [39], while a line point intercept method was used for basal cover of meadow vegetation Density by size class was measured with 60 m^2^ belt transects for shrubs and 1 m^2^ quadrats for herbaceous vegetation. For meadow vegetation, line point intercept transects were measured within each distinct meadow vegetation community in 2014. Basal hits were measured every 50 cm along 30 m transects to capture basal cover and percent composition.

### 2.4. Environmental Measurements

Air Temperature (°C) for the meadow and sagebrush steppe site was collected from the nearby SNOTEL site. SNOTEL sites are an automated system of snowpack and related climate sensors operated by the Natural Resource Conservation Service (NRCS). Soil Temperature (°C) was measured at the PJ site with eighteen ibutton temperature sensors (iBWetLand 22L, Alpha Mach, Bombardier, Ste-Julie, QC, Canada) located in and around the site, buried at 5 cm, and recording at 3 h intervals. Daily averages were calculated. Soil Volumetric Water Content (VWC) was measured using time domain reflectometry probes (CS 616, Campbell Scientific, Logan, UT, USA) within the upland sagebrush and PJ sites. CS616 probes were installed horizontally at a depth of 20 cm. Average daily VWC values were calculated from multiple soil probes for the upland sagebrush site (*N* = 12 probes) and the PJ site (*N* = 8 probes). Daily average VWC for the dry sagebrush site in the meadow was acquired from a nearby valley floor SNOTEL station, also located in a dry meadow community type, at a depth of 20 cm measured by a single probe (HydraProbe, Stevens Water Monitoring Systems, Portland, OR, USA). Two groundwater monitoring wells were used for this study, one in the mesic meadow and one in the wet meadow. Groundwater observation wells were installed in Porter Canyon in 2009. All wells were drilled using a Gidding’s auger fitted with a 4-in bit. A 2-in diameter polyvinyl chloride pipe perforated every 2 inches was placed in each auger hole to create the well. Water depth was measured using HOBO Water Level loggers (U20-001-01; ONSET Computer Corporation, Bourne, MA, USA) programmed to record hourly pressure. After correcting for atmospheric pressure, the values recorded by the HOBO loggers were converted to depth to water table. Hourly data was averaged to report daily water depth. All environmental daily data was used to calculate a 3-day moving average. The 3-day moving averages were used for all Figures and subsequent data interpretation. A 3-day moving average was used because it adequately smoothed the data, and because it was consistent with the 3-day moving window applied during the image filtering process described below.

### 2.5. Camera Setup

Cameras were configured and installed to view meadow, sagebrush steppe, and pinyon and juniper vegetation communities for a total of three separate camera sites, following the PhenoCam Network protocol [40]. Regions of interest (ROIs) for each site are illustrated in Figure 1 and details are listed in Table 1 and described below. Images used in this study were produced by StarDot NetCam SC 5MP IR-enabled cameras utilizing a complementary metal oxide semiconductor (CMOS) image sensor. Five images were taken per day at a resolution of 1296 × 960 pixels between 8 a.m. and 6 p.m. Cameras were mounted 2 m above ground and oriented for roughly 20% sky in the field of view (Figure 1). Due to the remote location of the study area standard data transmission techniques could not be utilized, therefore data was stored on compact flash cards and collected during field site visits every three months.

### 2.6. Image Data Processing

Initial analysis of image sets revealed that manual filtering was necessary due to differences in camera orientation, aspect and time of day. Our initial 10:00 a.m. to 6:00 p.m. or 8:00 a.m. to 4:00 p.m. time window with an image taken every two hours was too long and resulted in the sunlight angle causing over/under exposure and lens flare at specific times of day, which we decided to remove throughout the year leaving approximately three images per day. Blurred or weather obscured images were also removed. This initial analysis revealed clear patterns in lighting conditions that are attainable by using images from narrower time periods in subsequent years.

**Figure A1:**
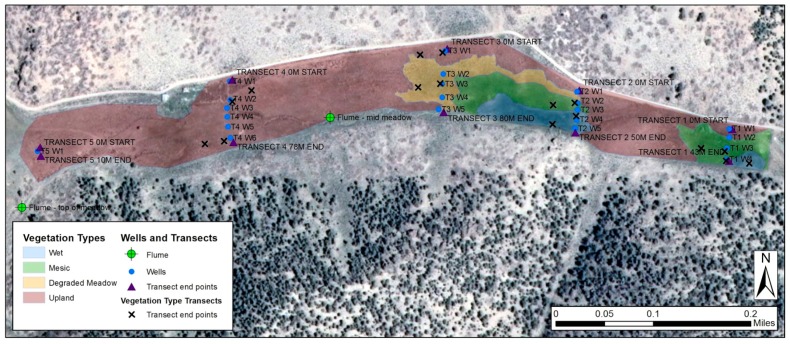
Porter Canyon meadow vegetation types and monitoring locations. Meadow phenocam focuses on the T2 transect of monitoring wells (see Figure 3 for alternate angle).

Image data was processed using the R *phenopix* package [37]. Regions of Interest (ROIs) were selected manually (i.e., manual visual selection of an area of interest) then drawn in R-studio using the *drawROI* function. The pixel coordinates were saved for later numerical analyses. Meadow site ROIs are representative of wet, mesic, and dry conditions according to previous research using a combination of gravimetric soil cores and vegetation survey mapping (K. A. Snyder and T. K. Stringham unpublished data [41]; Figure 1a and Figure A1). The dry region within meadow site was subset into a meadow—Sagebrush ROI that included only sagebrush canopy for direct comparison to the upland sagebrush canopy (Figure 1a). Upland sagebrush steppe ROIs were divided into canopy, interspace and an average for the whole community (Figure 1b). PJ canopy site consisted of three ROIs around three pinyon and three juniper individuals (Figure 1c).

**Figure 2:**
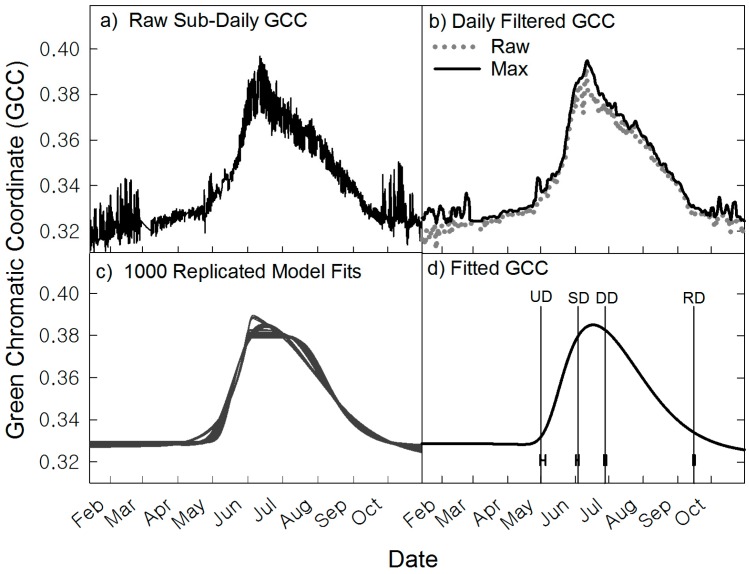
An example from the wet meadow of processing raw RGB values. (**a**) Raw GCC values acquired from extracting the per-pixel RGB digital numbers averaged across the ROI; (**b**) Three filters were applied in the order: *night, spline, max*. Max filtered GCC values calculated from sub-daily data after *night* and *spline* filters are applied. Raw GCC values at daily time steps are also shown as gray dots; (**c**) Filtered data fitted with uniform distribution of residuals around the observed data points to estimate uncertainty (1000 replications, gray lines); (**d**) The fitted line and median phenophase dates (vertical lines) upturn date (UD), stabilization date (SD), downturn date (DD) and recession date (RD) and the 10th to 90th confidence intervals around those dates.

**Figure 3:**
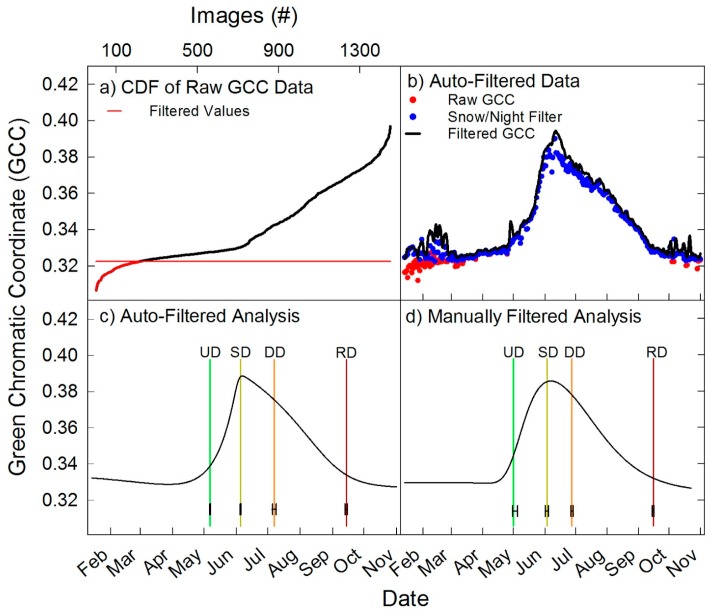
Analyses of the wet meadow to demonstrate snow/night filter. (**a**) Cumulative distribution frequency of GCC for all images, with the red line being the first break in slope and data below this was removed with snow/night filter; (**b**) raw data (red dots) with snow/night filter shown as blue dots, and black line is with the max filter applied; (**c**) Fitted and threshold seasonal GCC values obtained with the snow/night filter; (**d**) Fitted and threshold seasonal GCC values obtained on manually filtered images.

**Figure 4:**
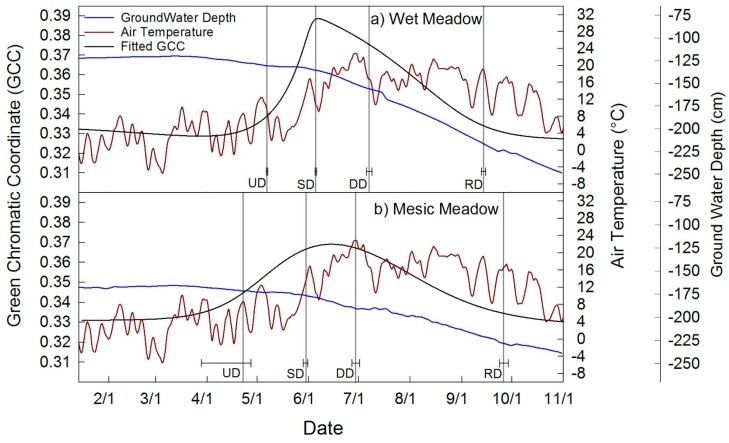
Seasonal course of GCC (black) and phenophase dates of (**a**) the wet meadow and (**b**) the mesic meadow, plotted with 3-day average daily air temperature (red) and average daily groundwater depth (blue).

**Figure 5:**
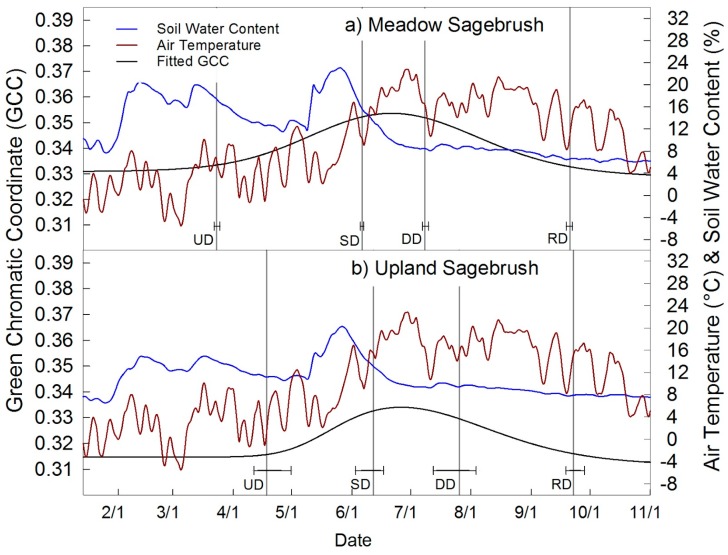
Seasonal course of GCC and phenophase dates are shown for (**a**) the dry meadow sagebrush canopy and (**b**) for the upland sagebrush canopy, plotted with 3-day average air temperature and soil water content. The dry meadow sagebrush had a longer growing season and increased greenness relative to the upland sagebrush canopy.

**Figure 6:**
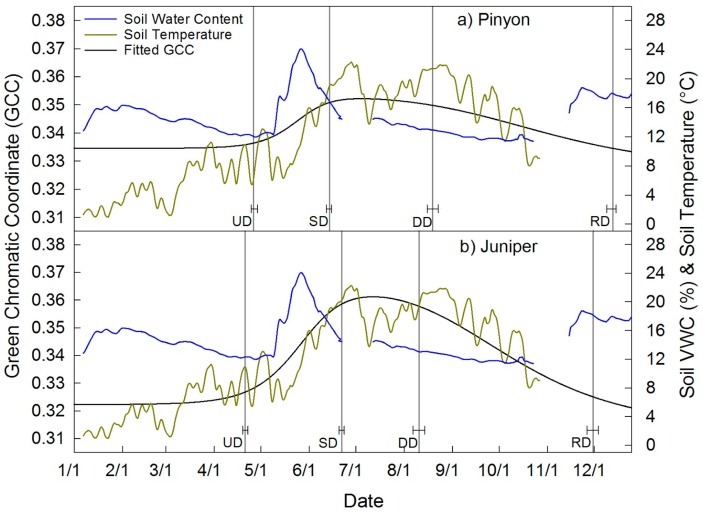
Seasonal course of GCC and phenophase dates for (**a**) pinyon and (**b**) juniper plotted with 3-day average daily soil temperature at 5 cm and soil volumetric water content (VWC). Note data gaps in soil VWC were caused by datalogger malfunction. Pinyon and juniper had a similar growing season. However, there was greater seasonal variation in greenness for juniper and a close tracking of green up with soil temperature in juniper.

**Figure 7:**
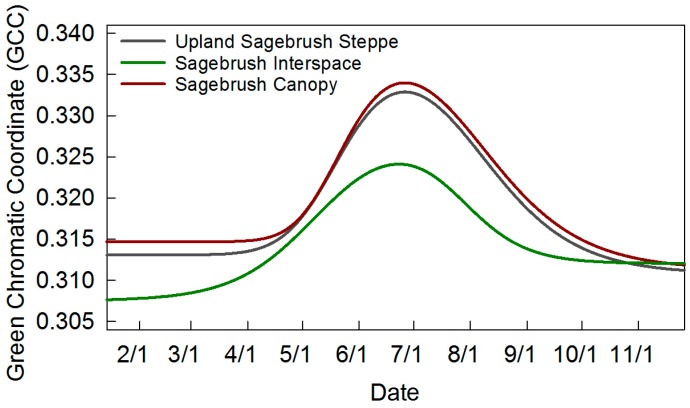
Fitted curves for 3 regions of interest (ROIs): sagebrush steppe community, sagebrush canopy, and interspace. ROIs are shown in Figure 1b.

Two methods were used for extracting DN values from ROIs, averaging across all pixels or retaining per-pixel values within an ROI. The first method results in a ROI-averaged GCC trajectory (as shown in Figure 2, Figure 3, Figure 4, Figure 5, Figure 6 and Figure 7). We first discuss the area-averaged method using data from the wet meadow as an example of data processing (Figure 2). First, Raw Red-Green-Blue digital numbers (DN) were extracted from each pixel within an ROI per image on a scale from 0 to 255 for each color channel. The data is then converted into a relative percent index and plotted as a time series, in this case the green chromatic coordinate (GCC):

(1) $$GCC=\frac{GDN}{\left(RDN+GDN+BDN\right)}$$

where the digital number (DN) of a given channel (R,G,B) is divided by the sum of all three channels (Figure 2a). Sub-daily GCC, red chromatic coordinate (RCC) and blue chromatic coordinate (BCC) values are then aggregated into daily values during the filtering process (Figure 2b). For the three ROI pinyon and juniper cases, sub-daily ROI values were averaged at each time step for the three individual canopies then filtered. Three filters were applied to sub-daily GCC datasets. The filters applied were “Night, Spline, Max” in that order [38] (p. 144). The *Night* filter removes low GCC values (below a GCC threshold of 0.2, resulting from poorly exposed images, i.e., dark, foggy or abnormal white balance images). The *spline-based* filter smooths the GCC values [42]. The *Max* filter computes the 90th percentile of GCC over a three-day moving window of GCC values resulting in a filtered time series at a daily temporal resolution [6].

Filtered data are then fitted with a double-logistic function following the formulation proposed by Gu et al. [43] (Figure 2c). A double logistic fit is an equation fitted to the data with a number of parameters to optimize to minimize the residuals between fitted and observed, for details of this fit see [38] (p. 144) and Gu et al. [43]. Other fitting and thresholding methods [44,45,46,47] available in the *phenopix* package were explored, but produced less consistent metrics or failed to process a phenophase in one or more of the ROIs, and therefore were discarded.

To determine the uncertainty of fitting (Figure 2c) and subsequent phenophase extraction, we used the method provided in *phenopix* [37] and described in Filippa et al. [38]. Briefly, for a given GCC time series the uncertainty function generates a uniform distribution of residuals around the observed data points. Double logistic fits are then applied recursively on data (1000 replications). For each replication, phenophases are extracted. The 10th and 90th percentile of the phenophase ensemble are then used as confidence intervals around the median phenophase dates, as shown in Figure 2d.

The Gu et al. [43] (pp. 42–46) method assigns four phenophases: an upturn date (UD) when GCC of vegetation begins to increase consistently, a stabilization date (SD) when vegetation approaches maximum GCC, a downturn date (DD) when GCC starts to consistently diminish, and a recession date (RD) when vegetation reaches a seasonal low (Figure 2d). Growing season length (GSL) was determined by subtracting UD from RD. Briefly, the maximum and minimum of the first derivative of each uncertainty fit is used to define the slopes of green up and senescence rates. A baseline and a max-line are also defined; these are two horizontal lines tangent to the minimum and the maximum. The intersection between these four lines defines the four phenophases in the original formulation by Gu et al. [43]. To account for the midseason decrease in GCC (the “greendown period” [45]) we have further defined a plateau line as a linear fit to values between SD and DD, in order to adjust the definition of phenophase DD. These phenophase determinations are based on the uncertainty analysis shown in panel c (Figure 2), not the fitted GCC line shown in panel d. An illustration of the above described phenophase methods is available in Filippa et al. [38].

**Figure 8:**
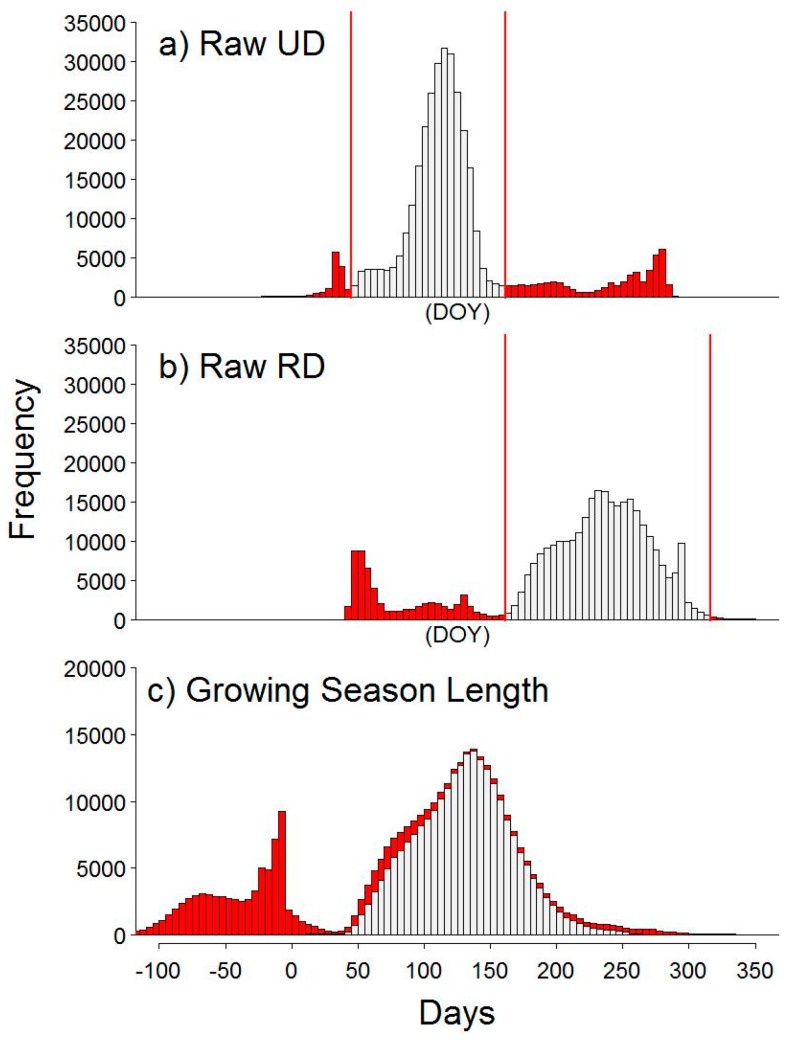
The manual filtering method used to determine pixels in the spatiotemporal analyses. Tick marks below bottom panel apply to all panels and are DOY for (**a**,**b**) and number of days for (**c**). (**a**,**b**) Vertical red lines represent lower and upper limits where pixels were removed due to bimodal distributions for upturn date (UD) and recession date (RD). Red bars are removed data. (**c**) Growing season length (GSL) calculated as the difference between RD and UD. Red bars illustrate pixels that characterized interspace and gray bars are pixels that characterized vegetation and were predominately sagebrush canopy. These data are used in the analyses in Figure 9.

**Figure 9:**
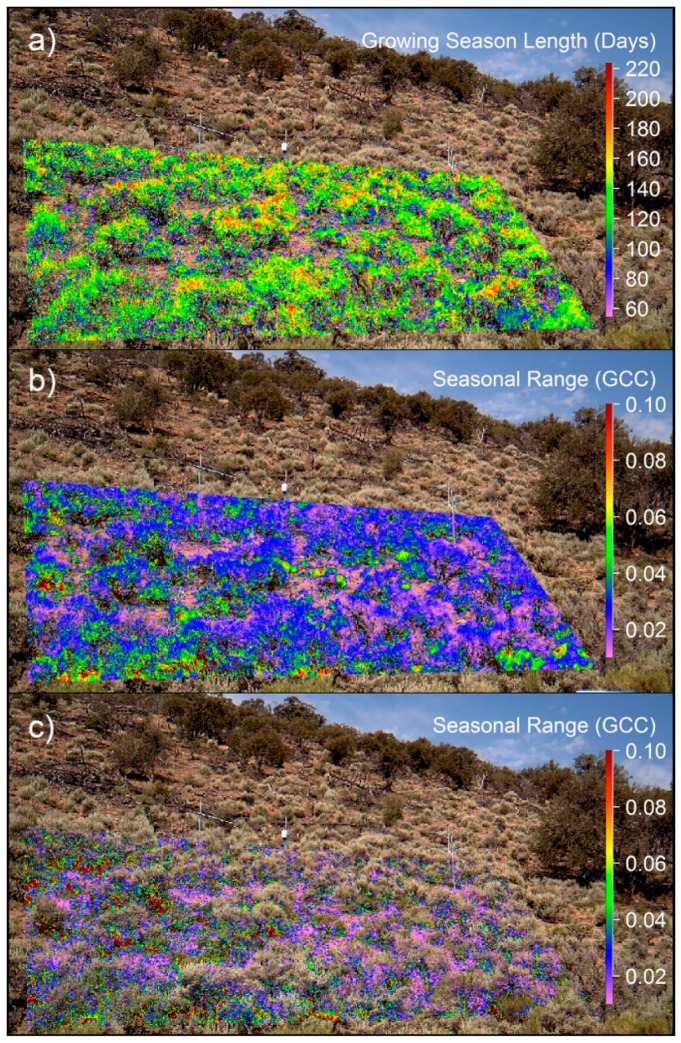
(**a**) Growing season length (GSL) illustrated for pixels of vegetation, data are from the gray bars in Figure 8c; (**b**) Seasonal GCC range of vegetation, pixels of vegetation were determined from the gray bars in Figure 8c; (**c**) Seasonal GCC range of the interspace, interspace pixels were determined from red bars in Figure 8c. Interspace pixels were as follows: green to red match bark and shadows and violet to blue match bare ground. (**b**,**c**) scales were kept fixed from 0.01–0.1 to highlight the two distributions.

The second method retains per-pixel values within a ROI and computes a GCC trajectory for each pixel, fits a curve to the data and extracts phenophases, resulting in a spatially-explicit phenophase map (as shown in Figure 8 and Figure 9). This pixel-by-pixel method will hereafter be called the *spatiotemporal* analysis for its ability to resolve patterns within a discrete area through time. The spatiotemporal analysis was performed by fitting a spline rather than a double logistic function, due to the frequent failure of the double logistic fitting with the spatial data. Uncertainty was not computed in the spatiotemporal analysis, because this was computationally demanding. The time series of GCC over a single pixel are noisier than those computed by averaging an entire ROI. Shadows and small camera shifts are responsible for the noisy signal that prevented the use of a double logistic approach for the pixel by pixel analysis. Spatial phenophase dates were determined with the Gu et al. [43] method used in the ROI-averaged approach. This spatiotemporal analysis was applied to the upland sagebrush steppe imagery.

### 2.7. Automated Snow vs. Manual Snow Filtering

**Figure A2:**
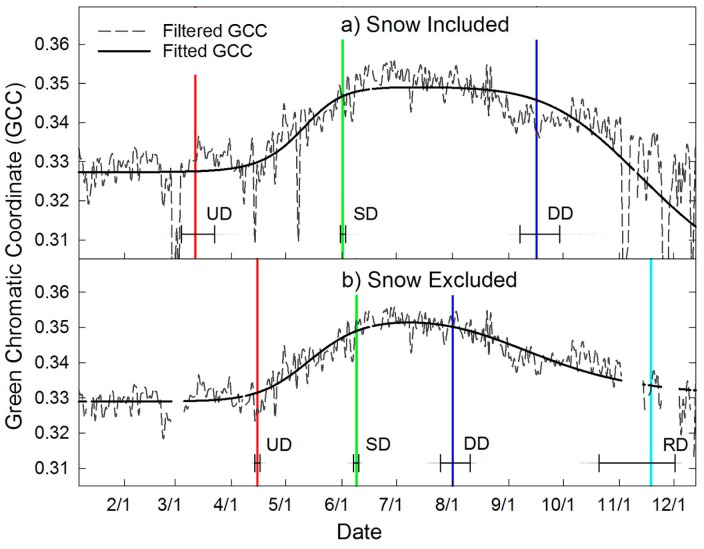
Phenology analyses of an individual pinyon canopy. (**a**) Image set contained all filtered images, including images with snow; (**b**) Image set contained the same images as (**a**), with all snow images removed.

Ephemeral snow pulses characterized the 2015 calendar year, which produced noise in GCC values and poor fits, or in the case of pinyon failure to fit a phenophase (data not shown; Figure A2). With snowy images included, November, December and January GCC values often showed downward trends and did not flatten out for dormancy (RD). Therefore, we explored refining our analyses by manually removing snow images before raw DN extraction (Step 1: Figure 2a). Snow images were determined as “any image of snow within or near a region of interest”. However, manually removing images is time consuming and subject to human bias. Therefore, we developed a method to automate removal of most snow images using the night filter by placing a varying lower limit on the GCC data per ROI, thus creating a variable night filter. We noted that the occurrence of unreasonably low GCC values, due to the presence of snow, resulted in a divergence from linearity of the cumulative distribution frequency (CDF) of GCC in its lower range. We therefore used a breakpoint approach (*strucchange* R package [48]), which finds when a linear regression becomes unstable and turns into a different stable linear regression. For each ROI’s raw data, the breakpoint between the first and second regression was then used in the snow/night filter and applied in the order described in Section 2.6. The process is illustrated in Figure 3a,b. We compared automatically filtered to manually filtered results and found them to be in good agreement. The results of the Gu fitting with the automated snow/night filter (Figure 3c) are shown in comparison to results obtained on the manually sorted images (Figure 3d). The fitted curves changed slightly with the two different methods, but the assignation of UD, SD and RD were very similar; DD was the exception shifting the median by nine days. The automated filter caused the fitted curve to more closely match the unfitted data by retaining a shallower post-season slope. Since the steepest downward slope is used in threshold calculation, a late season drop off resulted in a delayed DD assignation. However, the positioning of phenophases is based on the uncertainty estimation, not the curve fitting, which produced good agreement in dates between the two methods. The new snow/night filter was used for all the averaged ROI analyses. For the spatiotemporal analyses of the upland sagebrush we used the manually removed snow image set. Automated and manual snow filtering methods caused images to be removed in winter, spring and fall, but mostly from November to February. Manual filtering resulted in using 809 out the possible 1028 images in the spatiotemporal analyses, which resulted in 253 out of 365 days for the upland sagebrush site.

## 3. Results

### 3.1. Community Structure

Field surveys determined the dominant species within the ROIs. The upland PJ canopy site had 9.5% juniper and 32.3% pinyon canopy cover. The next dominant species was curl-leaf mountain mahogany (*Cercocarpus ledifolius* Nutt.) at 7.2%. Bare ground, litter and rock composed 62.6% of the understory with the remaining 37.4% dominated by Sandberg bluegrass (*Poa secunda*).

The upland sagebrush steppe and meadow sagebrush sites occur on hillslopes and valley bottoms surrounding the meadow and were dominated by mountain big sagebrush (*Artemisia tridentata subsp. vaseyena*) with an average canopy cover of 29.4%. Three additional shrub species: yellow rabbitbrush (*Chrysothamnus viscidiflorus* (Hook.) Nutt.), rubber rabbitbrush (*Ericameria nauseosa* (Pall. ex Pursh) G.L. Nesom & Baird) and spineless horsebrush (*Tetradymia canescens* DC.) also occurred within this plant community and together comprised 2.4% of the canopy cover. Total interspace area between shrubs comprised 68.2% of the upland sagebrush steppe site. Bare ground and litter exceeded 85% in the meadow sagebrush understory.

Composition of the wet meadow was 66.7% Nebraska sedge (*Carex nebrascensis)* an obligate wetland species. The mesic meadow community is 57.1% Douglas’ sedge (*Carex douglasii* Boott). Common yarrow (*Achillea millefolium* L.), Rocky Mountain iris (*Iris missouriensis* Nutt.), and prickly lettuce (*Lactuca serriola* L.) each made up 14.3% of the mesic community.

### 3.2. Averaged ROIs

The total precipitation for the water year 2015 was below the long-term average at 284.5 mm measured at the PCEW SNOTEL [49] station. The Porter Canyon winter of 2015 was warm and dry with a lack of consistent snowpack. Ephemeral pulses of snow occurred from January to May with an average event depth of 5.32 cm. However, 1 November to 31 December was less ephemeral with 89% days of snow cover at an average depth of 10.06 cm [49]. Removal of snow images with the automated snow/night filter increased the successful fitting of all four phenophases and the confidence of pinpointing phenophases (i.e., smaller confidence intervals) (Figure 3 and Figure A2).

Time series of GCC extracted from the wet and mesic meadow are shown in Figure 4, together with 3-day average air temperature and daily average water table depth (Figure 4). The wet meadow had a slightly later UD, day of year (DOY) 127 ± 0.5, and shorter total growing season 130 ± 1 days than the mesic meadow at 157 ± 14, respectively (Table 2). The wet meadow had greater annual GCC amplitude, with lower baseline greenness and a greater peak season (Table 2). The mesic meadow had a longer growing season but smaller annual GCC amplitude. More uncertainty was associated with the mesic UD (30 days). The stabilization dates (SD) and downturn dates (DD) were very similar. The wet meadow green up appeared to coincide with increased average temperature above 8 °C. Groundwater levels did not show the characteristic upturn in response to snowmelt, likely due to the fact that 2015 was the fourth year of below average precipitation. Groundwater levels declined throughout the growing season in both the mesic and wet meadow.

Time series of GCC extracted from the meadow sagebrush canopy and upland sagebrush canopy are shown in Figure 5, together with 3-day average air temperature and VWC at 20 cm (Figure 5). GCC for the dry meadow sagebrush canopy and the upland sagebrush canopy were less green and had lower seasonal amplitude than the wet and mesic meadow (Table 2, Figure 4 and Figure 5). The dry meadow sagebrush had a month longer growing season at 181 ± 2 days in comparison to the upland sagebrush canopy 157 ± 14 days, and may have initiated green up in response to increased and greater soil VWC relative to the upland sagebrush site. Upland and dry meadow sagebrush ROIs had similar GCC seasonal amplitude (0.022 and 0.025). Max GCC was greater in the meadow sagebrush (0.354) than the upland (0.334) (Figure 5). Fitted phenophases were more uncertain for the upland sagebrush with increased uncertainty in GSL of ±14 days, while dry meadow sagebrush was ±2 days.

Time series of GCC extracted from the pinyon and juniper canopies are shown in Figure 6, together with 3-day average soil temperature and 3-day average VWC (Figure 6). Pinyon had a similar length of growing season to juniper, 230 ± 4 and 224 ± 4 days, respectively. However, juniper had greater amplitude in GCC over the growing season compared to pinyon, 0.040 versus 0.019. Throughout green up, juniper GCC tracked with surface soil temperature more closely than did pinyon (Figure 6).

For the upland sagebrush site, three averaged ROIs (Figure 1b) were drawn and processed in order to estimate the differences between sagebrush steppe community, sagebrush canopy and sagebrush interspace (Figure 7). The fitted curves had expected outcomes, where the sagebrush canopy had max GCC (data is identical to fitted line in Figure 5 for reference), interspace was the lowest, and the entire community was in between (Table 2, Figure 7). The sagebrush canopy had a marked phenological signal, with an increase from about 0.31 GCC in spring to a peak of 0.33 in summer (Figure 7). In contrast, the interspace GCC had an overall lower green signal and a smoother trajectory increasing earlier than the canopy, most of the interspace is bare or litter, but the presence of scattered grasses that green up earlier contributed to this signal (Figure 7).

### 3.3. Spatiotemporal Analyses

**Figure A3:**
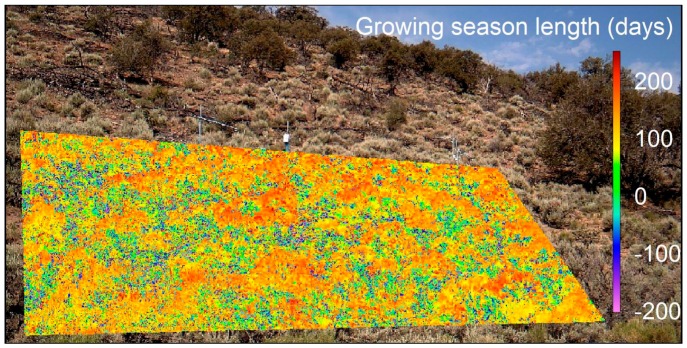
Initial spatiotemporal analysis that was confounded by interspace, which lead to the filtering described in Figure 8. The red and yellow colors closely map with sagebrush canopy, while green and blue closely map with interspace but have a negative growing season length.

Spatiotemporal analysis was performed on the upland sagebrush steppe imagery to identify growing season length (GSL) and seasonal GCC range at resolutions <9 cm^2^. Differences between sagebrush canopy and interspace were apparent; with the pixel by pixel analyses it became evident that differences between the bare interspace and green vegetation were producing erroneous negative values of GSL in the bare ground interspace, due to the weak and noisy signal of the interspace (Figure not shown; see Figure A3). We explored the possibility to discriminate these portions of the images by means of bifurcating the bimodal distributions shown in Figure 8a,b. Filtering was based on manual inspection of the frequency histograms of per-pixels values for UD and RD dates, and days of the year that fell outside the predominant bell-shaped distribution were removed (vertical red lines and red bars Figure 8a,b). The combined histogram was the calculated GSL (RD minus UD) (Figure 8c). The GSL distribution with negative values (shown in red) was caused by poorly fitted pixels that often resulted in UD thresholds (Red: Figure 8a) occurring after RD thresholds (Red: Figure 8b); which resulted in negative values (Red: Figure 8c). Data were excluded from UD data for DOY <50 and >170 and from RD data for DOY <170 and >330. The gray distributed GSL (Figure 8c) were plotted spatially in Figure 9a.

The median GSL for averaged upland sagebrush canopy was 157 ± 14 days (Table 2, Figure 5b), whereas the spatiotemporal analysis resulted in a median of 131 ± 48 days, and an interquartile range of 104 to 154 days. The spatiotemporal analysis was able to look at finer scale patterns of GSL, and the upper limits of the GSL range were similar to those of the spatially averaged ROI.

**Figure A4:**
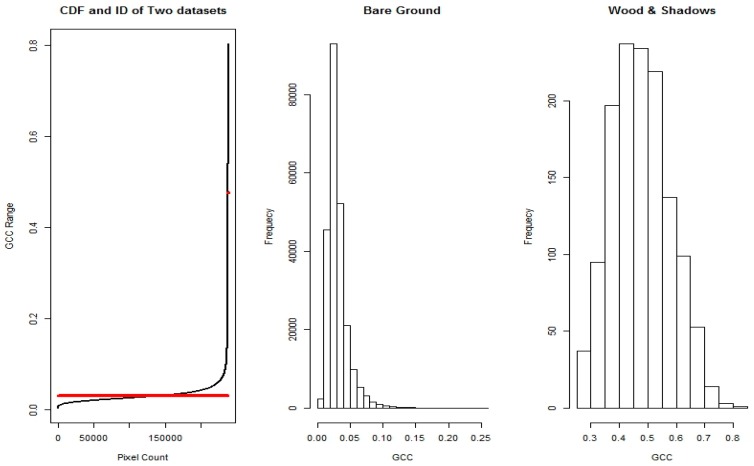
Cumulative distribution frequency (CDF) of data shown in Figure 9c, with histograms of the two ranges after splitting the original CDF.

To examine the spatial seasonal range of GCC, the difference between annual maximum GCC and minimum GCC was calculated for each individual pixel and plotted for both the shrub canopy (Figure 9b) and interspace groupings (Figure 9c). The groupings were determined from the frequency histogram in Figure 8c; therefore, grey bars represent pixels used in Figure 9a,b, while red bars represent data used in Figure 9c. Figure 9b,c scales were limited to a range of 0.01 and 0.1 for visual purposes. The shrub canopy (Figure 9b) had an average GCC range of 0.034 ± 0.017. For the interspace (Figure 9c), red to green colored pixels appeared to be located in woody and shaded areas when compared to the original images, while violet to blue pixels were bare ground. By ordering the data into a CDF, the method used in the snow/night filter, an R package called *changepoint* [50] split the interspace data by the mean GCC value (*cpt.mean*) and then divided the CDF into two histograms (data not shown; see Figure A4). We determined bare ground to have a median and 10th–90th percentile GCC range of 0.027 ± 0.016, while woody material and shadows were 0.472 ± 0.133.

## 4. Discussion

### 4.1. Methodological Considerations

Data presented in this paper demonstrate that the PhenoCam Network protocol, StarDot cameras and analyses with the *phenopix* package worked well to characterize plant phenology in the Great Basin, with some minor modifications. R’s *phenopix* package allows us to assess the best modeling method for comparing camera derived seasonal dynamics in Great Basin plant communities. Previous research utilizing phenocams focused on deciduous forests [16,17,18] and grasslands [22] (i.e., ecosystems with pronounced seasonal dynamics), resulting in a rather large green seasonal amplitude (on the order of 0.1–0.2 GCC) [17] (p. 105); whereas in this work, we demonstrate the applicability of phenocams in the retrieval of rather low phenological signals (e.g., 0.04 for juniper and even 0.01 for pinyon). Given this low phenological signal, we modified the standard data filtering procedure available in *phenopix* to remove images with ephemeral snow pulses and low GCC, in order to retrieve cleaner GCC seasonal trajectories with increased confidence intervals. For ROIs with high percentages of bare ground, we explored methods for extracting predictable vegetative information and differentiating vegetation from interspace (Figure 8, Figure 9 and Figure A3). This approach allowed us to focus on vegetation dynamics at <9 cm^2^ resolution.

The double-logistic formulation proposed by Gu et al. [43] was used to model seasonal GCC trajectories and extract phenophases. This formula was originally presented as a robust method for characterizing seasonal dynamics of canopy photosynthetic capacity (CPC), derived from net ecosystem exchange (NEE) measurements [51]. These data demonstrate the ability to apply this formula on vegetative data from camera images. We tested all available fitting methods in the *phenopix* package. Gu’s method for fitting and thresholding failed much less often than others [44,45,46], likely because the other double logistic formulations were primarily developed for satellite-based remotely sensed [44,45] or phenocam based [46] deciduous broad-leaved forests. Based on these findings, the formulation by Gu et al. [43] is potentially well-suited for monitoring grassland, perennial shrubland, and evergreen conifer temporal dynamics. A double logistic curve may not work for all semi-arid and arid environments, particularly in water-limited regions with a significant amount of monsoon rainfall characterized by a bi-modal growing season [52].

### 4.2. Meadow Phenology

Wet and mesic meadows showed slightly different phenologies. The magnitude of change in GCC was 0.06 and 0.04 for wet and mesic meadow, which is similar to the degree of change in GCC found in a fen, copse and heath areas in a low Arctic wetland area [53]. A later upturn date (UD) combined with an earlier recession date (RD) in the wet compared to the mesic meadow resulted in a roughly 27 days shorter growing season (Figure 4) at the wet meadow site. Additionally, the wet meadow appeared to respond more strongly to temperature relative to the mesic meadow. An emergence and survival study at a 2170 m elevation site found that average daily temperature had to exceed 10 °C in the spring for emergence of new shoots to begin [54]; this agrees very well with our finding (Figure 4a), which show meadow green up coincident with an increase in daily average temperature above 8 °C. There was greater uncertainty in the mesic meadow UD (±30 days). This was a result of the dry warm winter where some green up began in April and then stabilized; however, it appears late spring rains and warmer temperature accelerated green up in May.

The wet meadow is dominated by Nebraska sedge, which has a wide geographic and elevation range and is the only species present that is classified as an obligate wetland species [55]. This species is found in flat meadow areas where surface water flows episodically [54], but has also been shown to survive in field environments where depth to groundwater was 1.3 m [56]. Interestingly, depth to water in the wet meadow site at the beginning of green up was 130 cm, whereas it was 165 cm in the mesic meadow site. At the end of the growing season the two meadows exhibited similar depths to water with the wet meadow at 220 cm and the mesic meadow at 230 cm, respectively. The idea that the wet meadow community was responding to reduced water availability is plausible due to the two week earlier end of the growing season, when temperatures were still warm, but depth to groundwater was 220 cm. (Figure 4). The middle of the green-down occurred when depth to groundwater exceeded 150 cm.

### 4.3. Sagebrush Phenology

We were able to detect that the sagebrush canopy in the dry meadow was greener, had greater seasonal variation, and had a 24 day longer growing season than the drier upland sagebrush site, likely due to increased soil water content at 20 cm earlier in the winter and continuing into the spring. Soil water content was also greater in the meadow sagebrush than in upland sagebrush. The meadow sagebrush canopy began green up when soil moisture increased in early spring (3/23/15) that coincided with a brief and substantial increase in air temperature (Figure 5a). While the upland sagebrush initial green up was later (4/18/15) and did not appear to be driven by increased soil water content (Figure 5c). By analyzing spatially explicit phenological patterns (i.e., spatiotemporal analysis), coupled with a post processing of phenophase frequencies (Figure 8 and Figure 9) we were able to differentiate the sagebrush canopy and the interspace. Additionally, we could distinguish between interspace pixels of bare ground to woody material and shadows (Figure 9c). Subtle differences were seen in the sagebrush canopy. For example, the yellow to red pixels in Figure 9a correspond to the current year growth of shrub canopies, which are likely to have a longer growing season than previous year’s leaves. The larger change in GCC (Figure 9b) at the bottom of the ROI, shown in red and orange, was caused by the presence of rabbitbrush (*Ericameria nauseosa*), in a small portion of the ROI, demonstrating the potential utility of land-based spatiotemporal analysis to separate between shrub species. These data demonstrate the usefulness of the spatiotemporal analysis in showing intra- and inter-individual phenological differences. Similarly, a study by Julitta et al. [19], using spatial analysis in a subalpine grassland, found bimodality for beginning of season (BOS) dates in a year when there was a warming spell and early snowmelt. This bimodality in BOS was the result of pixels being dominated by two different vegetation types, grasses or forbs. Our approach can therefore facilitate the determination of areas of active growth in the sagebrush steppe and potentially other communities. The cameras used in this study have the capability to produce an NDVI (Normalized Difference Vegetation Index) estimate [15], which will further refine the estimated area of active growth.

### 4.4. Pinyon and Juniper Phenology

We were able to detect small phenological differences between evergreen pinyon and juniper canopies, with juniper canopies being characterized by a lower minimum and greater seasonal variation in GCC. Interestingly, the juniper curve began to rise as soil temperature began to increase after a late February and early March storm. A study examining the ability of two needle pinyon (*Pinus edulis*), a closely related species to singleleaf pinyon, and Utah juniper on their ability to use summer monsoon rainfall across a monsoon gradient found the two needle pinyon used less shallow soil water in mid-summer and postulated that this was due to high temperatures that limited surface root activity [57].

Rarely have phenocams used more than one region of interest [20]. Our PJ analyses averaged three separate individuals for each species by averaging data from three ROIs drawn around individual tree canopies. Subtle changes were evident when including more replication with the three ROI approach, such as an increased growing season length for juniper and a more dampened magnitude of seasonal GCC range for pinyon. Future work could examine the optimal number of ROIs, pixel area and/or individuals required for stabilizing date assignation of median phenophase transitions.

Studies using satellite derived NDVI in the Great Basin found that sagebrush steppe had minimal, yet detectable seasonal variation [58]. Cheatgrass exhibited extreme inter-annual NDVI variability in response to precipitation [59]. PJ had a less pronounced NDVI response than cheatgrass, but more pronounced than sagebrush steppe [23]. This agrees well with findings from the current study (Table 2), although we did not measure cheatgrass we did find the greatest variation in meadow dominated by sedges and grasses, similar to the findings for cheatgrass. Sagebrush had a muted GCC response in comparison to the wet and mesic meadows and juniper canopies. The one major difference was pinyon which had the least amount of seasonal GCC response unlike the findings of Bradley and Mustard [23]. This could be due to the fact that with land-based images we are able to distinguish between individual canopies of pinyon and juniper, while satellite derived indices have lower spatial resolution and therefore generally aggregate to the stand-level and include both species. One plausible explanation for the observed differences in the current study is that 2015 was the fourth year of below average precipitation at PCEW, and pinyon was showing signs of drought stress and damage from the bark beetle (*Ips* spp.). Although we tried to avoid choosing canopies that appeared damaged, it is possible that these algorithms detected damage not visible to the human eye.

### 4.5. Summary

In the Great Basin, climate has already exhibited warming of 0.3 °C to nearly 1.0 °C over the last century, and a decline in winter snowpack has been observed [60,61]. Given predicted increase of future temperature and decreases in snowpack [30], there is great a need to monitor associated plant phenology changes on scales relevant to land management decisions in the Great Basin. Due to the fragmented nature, both spatially and temporally, of phenological estimates taken by satellites and field collection, semi-continuous land-based camera phenology increases the information available. This facilitates examining phenology patterns at finer temporal and spatial scales, while allowing for more detailed cause and effect analysis with climate, soil water content, and other ecohydrological drivers. In the Great Basin, these cameras have a variety of potential applications. Management practitioners can make more informed decisions with regard to grazing periods and forage allocation. Knowledge of individual species phenological responses to varying climate will provide important information for drought management plans, post-fire rehabilitation seed mixes, grazing decisions and wildlife habitat manipulations. Cameras may provide monitoring of invasive species [62], such as cheatgrass which has a winter annual phenology. We have demonstrated that using PhenoCam Network protocols, StarDot cameras, and the *phenopix* package with modifications for ephemeral snow cover and bare ground, plant phenology can be effectively monitored over a range of communities in a cold desert environment.

## Figures and Tables

**Table 1:** Site locations used in this study within the Porter Canyon Experimental Watershed. Air temperature located at NRCS SNOTEL. Volumetric soil water content located at NRCS SNOTEL, upland sagebrush steppe, and pinyon and juniper sites. Soil temperature located at the pinyon and juniper site. Depth to groundwater well located at wet and mesic meadow region of interest.

Site	Latitude (dd)	Longitude (dd)	Area (m^2^)
Wet Meadow	39.46587 N	117.613008 W	300
Mesic Meadow	39.46605 N	117.612967 W	168
Meadow Sagebrush	39.46623 N	117.612894 W	110
Upland Sagebrush Steppe	39.46599 N	117.622114 W	528
Upland PJ Canopy	39.46333 N	117.623339 W	72 per spp.
NRCS SNOTEL	39.46542 N	117.620694 W	-

**Table 2:** A summary of phenophase dates from averaged regions of interest (ROI Sites): upturn date (UD), stabilization date (SD), downturn date (DD), recession date (RD), growing season length (GSL), annual minimum GCC, maximum GCC, and total range of green chromatic coordinates (GCC) with the associated figure number.

Site	UD	SD	DD	RD	GSL	Min	Max	Range	Figure
	DOY ± Days				Days	GCC			#
**Wet Meadow**	127 ± 0.5	156 ± 0.5	188 ± 2	257 ± 1	130 ± 1	0.327	0.389	0.062	4a
**Mesic Meadow**	113 ± 15	150 ± 1	180 ± 2	269 ± 3	157 ± 14	0.329	0.369	0.040	4b
**Meadow Sagebrush Canopy**	82 ± 1	157 ± 1	189 ± 2	264 ± 2	181 ± 2	0.329	0.354	0.025	5a
**Upland Sagebrush Canopy**	108 ± 10	163 ± 7	207 ± 11	265 ± 5	157 ± 14	0.312	0.334	0.022	5b, 7
**Upland Pinyon**	116 ± 2	165 ± 2	231 ± 4	347 ± 3	230 ± 4	0.333	0.352	0.019	6a
**Upland Juniper**	111 ± 2	173 ± 2	223 ± 4	334 ± 4	224 ± 4	0.321	0.361	0.040	6b
**Upland Sagebrush Steppe**	99 ± 22	167 ± 17	194 ± 22	269 ± 8	170 ± 28	0.311	0.333	0.022	7
**Upland Sagebrush Interspace**	119 ± 24	144 ± 14	223 ± 5	265 ± 10	146 ± 15	0.308	0.324	0.016	7

## Appendix A

Figures not shown in the main manuscript.
